# *In Situ* Characterization of Human Lymphoid Tissue Immune Cells by Multispectral Confocal Imaging and Quantitative Image Analysis; Implications for HIV Reservoir Characterization

**DOI:** 10.3389/fimmu.2021.683396

**Published:** 2021-06-09

**Authors:** Eirini Moysi, Perla M. Del Rio Estrada, Fernanda Torres-Ruiz, Gustavo Reyes-Terán, Richard A. Koup, Constantinos Petrovas

**Affiliations:** ^1^ Vaccine Research Center, National Institute of Allergy and Infectious Diseases, National Institutes of Health, Bethesda, MD, United States; ^2^ Centro de Investigación en Enfermedades Infecciosas, Instituto Nacional de Enfermedades Respiratorias, Mexico City, Mexico; ^3^ Comisión Coordinadora de Institutos Nacionales de Salud y Hospitales de Alta Especialidad, Secretaría de Salud, Mexico City, Mexico; ^4^ Institute of Pathology, Department of Laboratory Medicine and Pathology, Lausanne University Hospital, Lausanne, Switzerland

**Keywords:** lymph nodes, Tfh, confocal imaging, tissue architecture, HIV reservoir

## Abstract

CD4 T cells are key mediators of adaptive immune responses during infection and vaccination. Within secondary lymphoid organs, helper CD4 T cells, particularly those residing in germinal centers known as follicular helper T cells (Tfh), provide critical help to B-cells to promote their survival, isotype switching and selection of high affinity memory B-cells. On the other hand, the important role of Tfh cells for the maintenance of HIV reservoir is well documented. Thus, interrogating and better understanding the tissue specific micro-environment and immune subsets that contribute to optimal Tfh cell differentiation and function is important for designing successful prevention and cure strategies. Here, we describe the development and optimization of eight multispectral confocal microscopy immunofluorescence panels designed for in depth characterization and immune-profiling of relevant immune cells in formalin-fixed paraffin-embedded human lymphoid tissue samples. We provide a comprehensive library of antibodies to use for the characterization of CD4+ T-cells -including Tfh and regulatory T-cells- as well as CD8 T-cells, B-cells, macrophages and dendritic cells and discuss how the resulting multispectral confocal datasets can be quantitatively dissected using the HistoCytometry pipeline to collect information about relative frequencies and immune cell spatial distributions. Cells harboring actively transcribed virus are analyzed using an in-situ hybridization assay for the characterization of HIV mRNA positive cells in combination with additional protein markers (multispectral RNAscope). The application of this methodology to lymphoid tissues offers a means to interrogate multiple relevant immune cell targets simultaneously at increased resolution in a reproducible manner to guide CD4 T-cell studies in infection and vaccination.

## Introduction

To understand the tissue specific mechanisms that govern successful clinical outcomes in infection and vaccination, for example pathogen clearance or the production of neutralizing antibodies, it is important to understand the context in which these outcomes arise. Lymphoid tissues and secondary lymphoid organs (SLOs) are anatomical sites with a key role in the generation of adaptive immune responses (1). They are characterized by a high density of lymphocytes compared to peripheral blood (estimated at ~60% in SLOs compared to 2.2% in blood) (2, 3) and are also populated by cell subsets such as fibroblasts and stromal cells that perform critical immune related functions (4–7) but are less amenable to isolation for *ex vivo* study. Traditionally, the study of immune cell dynamics in lymphoid tissues has been performed using single-cell methodologies such as flow cytometry and more recently mass cytometry (i.e. CyTOF) and single-cell or bulk RNAseq after dissociating the cells from the tissue. Such methodologies have greatly enriched our understanding of circulating and tissue-resident immunophenotypes, their transcriptional programs and population kinetics at different life stages or in pathology (8–11). However, methodologies employing cell suspensions do not interrogate by design the microanatomical segregation or positioning of immune subsets relative to each other despite the importance of these two parameters for mechanism and function (12).

HIV infection profoundly affects the microarchitecture of lymphoid tissues and lymph nodes (LN) (13). The virus is detected in LN shortly after infection (14) where it persists for life, even in the context of antiretroviral therapy (13, 15). Structural damage of B-cell follicles, LN fibrosis as well as acute lymphadenitis characterized by focal hemorrhages, extensive cellular destruction, accumulation of neutrophils, phagocytosis of nuclear debris, proliferation of blood vessels, presence of immunoblasts and clear cell aggregation are some of the changes that have been described in individuals affected by the virus (13). The CD4 T cell lineage, which comprises several subsets including Th1, Th2, Th9, Th17, Th22, regulatory T cells (Treg) and follicular helper T cells (Tfh) (16, 17) is a major target of HIV infection (18). CD4 T cell lineage subsets show varying distributions in peripheral blood and lymphoid tissue compartments depending on their trafficking and functional profiles as well as status of differentiation (naïve; central memory Tcm; effector memory Tem; terminal effector Temra) (8, 11). Of particular interest among these subsets are Tfh, a CD4 T cell population that was first identified in tonsillar tissues, in microanatomical regions known as germinal centers (GC) that develop after antigenic challenge (4, 19). Tfh are phenotypically characterized by the expression of the transcriptional regulator Bcl-6 and chemokine receptor CXCR5, as well as by the concurrent expression of the costimulatory receptors PD-1, CD40L and ICOS (4, 20). Their role is to provide critical help to B-cells to promote their survival, isotype switching and selection of high affinity memory B cells (21). In line with the heterogeneity observed in other CD4 T-cell subsets, Tfh also display phenotypic heterogeneity that has been shown to associate with function (21, 22). Furthermore, the exact topology of Tfh within GCs is also emerging as an important dimension for their analysis (23, 24) HIV infection has been shown to perturb Tfh numbers both in humans as well as in NHP animal models of the disease (25, 26). In addition, Tfh have been shown to represent an important reservoir of HIV during chronic infection (27, 28). Understanding thus the changes that come about in lymphoid tissues during a chronic infection is important for appreciating the mechanisms leading to pathology, disease progression as well as neutralizing – and broadly neutralizing- antibody formation.

In this study, we describe the development of eight multispectral confocal imaging panels designed for the analysis of CD4+ T-cells -including Tfh and regulatory T-cells- as well as CD8 T-cells, B-cells, macrophages and dendritic cells in tissue sections. Two additional panels designed for the characterization of key lymphoid stromal elements and HIV RNA positioning are also presented. We discuss how to prepare the confocal microscope for image acquisition, provide a comprehensive list of validated antibodies to use and suggest a framework for the quantitative analysis of the imaging results using the HistoCytometry pipeline (29). The proposed tissue analysis can be applied in studies of natural HIV infection as well as in experimental vaccination protocols to supplement existing datasets with spatial information to aid the generation of hypothesis-driven questions regarding the mechanisms underlying successful immune responses.

## Materials and Methods

### Ethics Statement

Signed informed consent was obtained before all procedures in accordance with the Declaration of Helsinki and approved by the appropriate Institutional Review Board. Tonsils were obtained from anonymized discarded pathologic specimens from Children’s National Medical Center (CNMC) under the auspices of the Basic Science Core of the District of Columbia Developmental Center for AIDS Research. The CNMC Institutional Review Board determined that study of anonymized discarded tissues did not constitute ‘human subjects research’. The inguinal lymph nodes used for the RNAscope and for LN-specific validations were from HIV+ donors recruited at Centro de Investigación en Enfermedades Infecciosas (CIENI), Instituto Nacional de Enfermedades Respiratorias (INER) in Mexico City, Mexico. Informed consent was obtained before the procedures in line with the protocols approved by the INER-CIENI Ethics Committee.

### Tissue Processing

Upon receipt of tissue, lymph nodes and tonsils were washed with ice-cold medium R-10 (RPMI 1640 supplemented with 10% fetal bovine serum, 2 mM L-glutamine, 100 U/mL penicillin and 100 µg/mL streptomycin (Invitrogen). The surrounding fatty tissue was removed, and tissues were cut into small pieces and immediately placed in fixative for 24hrs that was either in 10% Neutral Buffered Formalin (tonsils) or 4% neutral buffered paraformaldehyde for RNAscope as preliminary experiments showed superior results using this type of fixation (lymph node samples). Specimens were then embedded in paraffin and sectioned for multiplex confocal microscopy analysis. Tonsillar mononuclear cells used in flow cytometry experiments were isolated from specimens by mechanical disruption and subsequent Ficoll-Paque density gradient centrifugation.

### Antibodies

The following antibodies were used in the study (also summarized in **Table 1**).

Table 1Antibodies used for confocal imaging and flow cytometry.

**Table:** 

Confocal Imaging	Fluorochrome	Clone	Catalog Number	Source	Dilution
Biomarker					
**Bcl-6**	Unconjugated	PG-B6p	M7211	DAKO	1:100
**Bcl-6**	Unconjugated	LN22	MAB9607	Abnova	1:50
**Ki67**	Brilliant Violet 421	B56	562899	BD Horizon	1:50
**Ki67**	Brilliant Violet 480	B56	566109	BD Horizon	1:50
**Ki67**	Alexa Fluor 647	B56	558615	BD Biosciences	1:50
**PD-1**	Alexa Fluor 488	polyclonal	FAB7115G	R&D Systems	1:20
**CD20**	eFluor 615	L26	42-0202-82	eBioscience	1:50
**CD57**	Brilliant Violet 480	NK-1	555618	BD Horizon	1:50
**FoxP3**	Alexa Fluor 647	206D	320114	Biolegend	1:10
**CD4**	Alexa Fluor 700	polyclonal	FAB8165N	R&D Systems	1:50
**CD138**	Unconjugated	Ep201	BSB6530	BioSB	1:200
**CD38**	Unconjugated	SPC32	BioSB6201	BioSB	1:200
**H2Ax**	Alexa Fluor 647	N1-431	560447	BD Biosciences	1:10
**Helios**	Unconjugated	polyclonal	GTX115629	GeneTex	1:20
**CD25**	Unconjugated	4C9	BSB6320	BioSB	1:10
**IL-10**	Alexa Fluor 546	E-10	Sc-8438 AF546	Santa Cruz	1:10
**MPO**	Unconjugated	polyclonal	A0398	Agilent-DAKO	1:300
**CD68**	Unconjugated	KP-1	M0814	Agilent-DAKO	1:200
**CD163**	Alexa Fluor 647	EDHu-1	NB110-40686AF647	Novus	1:100
**FasL**	Unconjugated	polyclonal	Ab134401	Abcam	1:10
**CD8**	Unconjugated	4B11	4B11	Invitrogen	1:100
**Granzyme B**	Unconjugated	GrB-7	M7235	DAKO	1:100
**CD11c**	Unconjugated	2F1C10	60258-1-IG	ProteinTech	1:200
**CD123**	Unconjugated	BSB-59	BSB5327	BioSB	1:50
**CLEC9A**	Unconjugated	polyclonal	ab223188	Abcam	1:10
**CD31**	Unconjugated	polyclonal	ab28364	Abcam	1:10
**CD20**	Unconjugated	L26	14-0202-82	eBioscience	1:50
**IgD**	Alexa Fluor 488*	EPR6146	ab124795	Abcam	1:10
**FDC**	Unconjugated	CNA.42	F3803	Sigma	1:200
**Collagen I**	Unconjugated	3G3	LS-B5932	LSBio	1:500
**Collagen IV**	Unconjugated	LS-B16212	LS-B16212	LSBio	1:500
**CD3**	Unconjugated	F7.2.38	M7254	DAKO	1:300
**CD4**	Unconjugated	polyclonal	ab133616	Abcam	1:10

**Table:** 

Flow Cytometry	Fluorochrome	Clone	Catalog Number	Source
Biomarker				
**CD3**	BB700	SP34-2	566518	Beckton Dickenson
**Ki67**	Brilliant Violet 241	B56	562899	BD Horizon
**CD3**	H7APC	SK7	641406	BD Biosciences
**CD8**	Pacific Blue	RPA-T8	558207	BD Pharmingen
**CD20**	Brilliant Violet 570	2H7	302332	Biolegend
**CD4**	Brilliant Violet 650	SK3	563875	BD Biosciences
**PD-1**	Brilliant Violet 711	EH12.2H7	329928	Biolegend
**Bcl-6**	PE	K112-91	561522	BD Pharmingen
**CD57**	Alexa Fluor 594	NK-1	NBP2-47789AF594	Novus Biotechne
**CXCR5**	Cy7PE	MU5UBEE	25-9185-42	Thermo Fisher
**CD19**	FITC	J3-119	IM1284U	Beckman-Coulter
**CD38**	Brilliant Violet 786	HIT2	303530	Biolegend
**IgG**	BUV395	G17-1	104385	BD Biosciences
**H2Ax**	Alexa Fluor 647	N1-431	560447	BD Biosciences

### Flow Cytometry

CD3 BB700 (clone SP34-2, Beckton Dickenson) or CD3 H7APC (clone SK7, BD Biosciences); Ki67 Brilliant Violet 421 (clone B56, BD Horizon); CD20 Brilliant Violet 570 (clone 2H7, Biolegend); CD4 Brilliant Violet 650 (clone SK3, BD Biosciences); PD-1 Brilliant Violet 711 (clone EH12.2H7, Biolegend); Bcl-6 PE (clone K112-91, BD Pharmingen); CD57 Alexa Fluor 594 (clone NK-1, Novus Biotechne); CXCR5 (CD185) Cy7PE (clone MU5UBEE, Thermo Fisher); CD8 Pacific Blue (clone RPA-T8, BD Pharmingen); CD19 FITC (clone J3-119, Beckman-Coulter); CD38 Brilliant Violet 786 (clone HIT2, Biolegend); IgG1 BUV395 (clone G17-1, BD Biosciences); H2AX (pS139) Alexa Fluor 647 (clone N1-431, BD Pharmingen).

### Imaging

#### Primary/Conjugated

*Tfh panel:* Bcl-6 unconjugated (clone PG-B6p, DAKO); Ki67 Brilliant Violet 421 (clone B56, BD Horizon); PD-1 Alexa Fluor 488 (goat polyclonal, R&D systems); CD20 eFluor 615 (clone L26, eBioscience); CD57 Brilliant Violet 480 (clone NK-1, BD Horizon); FoxP3 Alexa Fluor 647 (Clone 206D, Biolegend); CD4 Alexa Fluor 700 (goat polyclonal, R&D systems) *B-cell panel:* CD138 unconjugated (clone Ep201, BioSB); CD38 unconjugated (clone SPC32, BioSB); Bcl-6 unconjugated (clone LN22, Abnova); Ki67 Brilliant Violet 480 (clone B56, BD Horizon); CD20 eFluor 615 (clone L26, eBioscience); H2Ax Alexa Fluor 647 (clone N1-431, BD Pharmingen); CD4 Alexa Fluor 700 (polyclonal, R&D systems) *Regulatory T-cell panel:* Helios unconjugated (polyclonal, GeneTex); CD25 unconjugated (clone 4C9, BioSB); Ki67 Brilliant Violet 480 (clone B56, BD Horizon); CD20 eFluor 615 (clone L26, eBioscience); FoxP3 Alexa Fluor 647 (clone 206D, Biolegend); IL-10 Alexa Fluor 546 (clone E-10, Santa Cruz); CD4 Alexa Fluor 700 (polyclonal, R&D systems) *Inflammation panel:* MPO unconjugated (polyclonal, Agilent-DAKO); CD68 unconjugated (KP-1, Agilent-DAKO); PD-1 Alexa Fluor 488 (goat polyclonal, R&D systems); CD20 eFluor 615 (clone L26, eBioscience); Ki67 Brilliant Violet 480 (clone B56, BD Horizon); CD163 Alexa Fluor 647 (clone EDhu-1, Novus); CD4 Alexa Fluor 700 (polyclonal, R&D systems) *CD8 T cell panel:* FasL unconjugated (polyclonal, Abcam); CD8 unconjugated (4B11, Invitrogen); Granzyme B (GrB-7, DAKO); CD20 eFluor 615 (clone L26, eBioscience); CD57 Brilliant Violet 480 (clone NK-1, BD Horizon); CD4 Alexa Fluor 700 (polyclonal, R&D systems);Ki67 Alexa Fluor 647 (B56, BD Biosciences) or for the *alternative CD8 T cell panel:* FasL unconjugated (polyclonal, Abcam); CD8 unconjugated (4B11, Invitrogen); Granzyme B (GrB-7, DAKO); CD3 (F7.2.38, Dako); CD20 eFluor 615 (clone L26, eBioscience); Ki67 Brilliant Violet 480 (clone B56, BD Horizon); CD4 Alexa Fluor 700 (polyclonal, R&D systems); *DC panel:* CD11c unconjugated (clone 2F1C10, ProteinTech); CD123 unconjugated (clone BSB-59, BioSB); CLEC9A (polyclonal, Abcam); Ki67 Brilliant Violet 480 (clone B56, BD Horizon); CD20 eFluor 615 (clone L26, eBioscience); CD4 Alexa Fluor 700 (polyclonal, R&D systems); CD8 unconjugated (4B11, Invitrogen); CD8 unconjugated (4B11, Invitrogen) *Structure panel:* CD31 unconjugated (polyclonal, Abcam); CD20 unconjugated (L26, eBioscience); IgD Alexa Fluor 488 (polyclonal, R&D systems – custom made on AF488), FDC unconjugated (CNA.42, Sigma); Collagen I (3G3, LSBio); Collagen IV (LS-B16212; LSBio); Ki67 Brilliant Violet 480 (B56, BD Horizon).

#### Secondary

*Tfh panel:* Goat anti-Mouse IgG1 Alexa Fluor 546 (A21123, Life Technologies); *B-cell panel:* Donkey anti-rabbit Brilliant Violet 421 (poly4064,406410, Biolegend); Goat anti-mouse IgG1 Alexa Fluor 546 (A21123, Life Technologies); Goat anti-mouse IgG2b Alexa Fluor 488 (A21141, Thermo Scientific) *Regulatory T-cell panel:* Donkey anti-rabbit Brilliant Violet 421 (poly4064, 406410, Biolegend); Goat anti-mouse IgG2b Alexa Fluor 488 (A21141, Thermo Scientific) *Inflammation panel:* Donkey anti-rabbit Brilliant Violet 421 (poly4064, 406410, Biolegend); Goat anti-mouse IgG1 Alexa Fluor 546 (A21123, Life Technologies) *CD8 T cell panel:* Donkey anti-rabbit Brilliant Violet 421 (poly4064, 406410, Biolegend); Goat anti-mouse IgG2b Alexa Fluor 488 (A21141, Thermo Scientific); Goat anti-mouse IgG2a Alexa Fluor 546 (A21133, Thermo Scientific) *Alternative CD8 T cell panel:* Donkey anti-rabbit Brilliant Violet 421 (poly4064, 406410, Biolegend); Goat anti-mouse IgG2b Alexa Fluor 488 (A21141, Thermo Scientific); Goat anti-mouse IgG2a Alexa Fluor 546 (A21133, Thermo Scientific); Goat anti-mouse IgG1 Alexa Fluor 647 (A-21240, Thermo Scientific). *DC panel:* Donkey anti-rabbit Brilliant Violet 421 (poly4064, 406410, Biolegend); Goat anti-mouse IgG2a Alexa Fluor 488 (A-21131, Thermo Scientific); Goat anti-mouse IgG1 Alexa Fluor 546 (A-21123, Thermo Scientific); Goat anti-mouse IgG2b Alexa Fluor 647 (A-21242, Thermo Scientific) *Structure panel:* Donkey anti-rabbit Brilliant Violet 421 (poly4064, 406410, Biolegend); Goat anti-mouse IgG2a Alexa Fluor 546 (A-21133, Thermo Scientific); Goat anti-mouse IgM Alexa Fluor 647 (115-607-020, Jackson ImmunoResearch); Goat anti-mouse IgG3 Alexa Fluor 594 (A-21155, Thermo Scientific); Goat anti-mouse IgG1 Alexa Fluor 594 (A-21125, Thermo Scientific).

### Flow Cytometry

Tonsil cells (1-2 x 10^6^) were stained with the Aqua Live/Dead Viability Dye (Invitrogen) and titrated amounts of antibodies against CD3, CD20, CD4, CD8, PD-1, CD57 and CXCR5 or CD3, CD8, CD19, CD38, IgG and CD20. ICS staining for Ki67 and Bcl-6 or H2Ax was performed using the BD Cytofix/Cytoperm Kit (BD Biosciences. At the end of the protocol cells were fixed with 1% parafolmaldehyde and acquired on a BD FACSymphony Flow Cytometer (BD Biosciences) running the BD FACSDiva software.

### Immunofluorescent Tissue Staining

Tonsillar tissue was used in seven out of the eight panels described (GC reactivity, T-cell regulation, B-cell immunity, CD8 T-cell positioning and function, monocyte and inflammatory markers, GC microarchitecture, tissue-specific DC subpopulations). Confocal imaging of tonsil sections (~10um in thickness) was performed using formalin fixed paraffin embedded (FFPE) tissues. Tissue sections were baked at 60°C for 1 hour and deparaffinized by serial immersions in xylene and ethanol dilutions. Antigen retrieval was performed at 110°C for 15 minutes using Borg RTU (Biocare Medical). Tissue sections were blocked and permeabilized for 1 hour at room temperature (RT) (1M Tris, 0.3% Triton X-100, 1% Bovine Serum Albumin) and stained with titrated amounts of antibodies. Stainings were carried out consecutively with the primary antibodies being added first and incubated overnight at 4°C, followed by staining with the appropriate secondary antibody. Conjugated antibody stainings were performed for 2 hours at RT, after which sections were stained with JOPRO-1 Iodide (Invitrogen) for nucleus identification and mounted with Fluoromount G (SouthernBiotech).

### Multispectral RNAscope

vRNA+ detection was based on specimens originating from HIV+ individuals (**Table 2**). For this purpose, formalin-fixed, paraffin-embedded lymph node tissue sections (~10um thick) were stained with HIV RNA probes (Cat No. 416111) using the RNAscope Multiplex Fluorescent Reagent Kit v2 (ACD) according to the manufacturer’s instructions. Briefly, sections were baked for 1 hour at 60°C and deparaffinized using serial xylene and ethanol baths. This was followed by an antigen retrieval step performed at 100°C for 15 minutes and a proteinase K treatment step for 20 minutes at 40°C. Subsequently, sections were incubated with HIV-1 Clade B-specific RNA probes (ACD) at 40°C for 2 hours in a HybEz hybridization oven (ACD) before being subjected to 4 rounds of signal amplification performed using the RNAscope amplification reagents. To visualize the RNA signal a tyramide-based detection system was used (TSA Plus Cyanine 5, Akoya Biosciences) per manufacturer’s instructions. To ascertain the specificity of the vRNA+ signal a modified version of the RNAscope protocol was used in which tissues were pretreated with 150ul of RNase A/T1 mix in 1x PBS (RNase cocktail Enzyme mix, 500U/ml A; 20.000 U/ml T1, Thermo Fisher) for 30mins at 40°C prior to target probe hybridization. For CD4+ T cell subset and FDC visualization sections were stained with antibodies against CD3 (F7.2.38, Dako), CD4 (rabbit polyclonal, abcam) and FDC (CNA.42, Sigma) at the end of the RNAscope protocol. The following secondary antibodies were used for CD3, CD4 and FDC visualization: Goat anti-Mouse IgG1 Alexa Fluor 546 (A21123, Life Technologies), Donkey anti-Rabbit Brilliant Violet 421 (406410, Biolegend) and Goat anti-Mouse IgM Alexa Fluor 594 (A21125, Life Technologies).

**Table 2:** Demographic and clinical details of study participants.

Experiment	Tissue ID	Age	Gender	VL (copies/uL)	CD4 count	Classification
Bcl-pH2Ax	HIV-1 #1	28	M	91357	261	Tx Naïve, Chronic
Bcl-pH2Ax	HIV-1 #2	34	M	84687	142	Tx Naïve, Chronic
RNAscope_main	HIV-1 #3	23	M	1,205,036	315	Tx Naïve, Chronic
RNAscope_controls	HIV-1 #4	19	M	621,622	290	Tx Naïve, Chronic
CD8-FasL	HIV-1 #5	31	M	67,764	721	Tx Naïve, Chronic

### Confocal Imaging and Analysis

Images were acquired on a Leica TCS SP8 confocal platform running LAS-X at a 512 x 512 pixel density with a 1x optical zoom using a 40x objective (NA 1.3) unless otherwise stated. The microscope was equipped with 3 HyD and 2 PMT detectors and 11 laser lines (405, 457, 476, 488, 496, 514, 561, 594, 633, 685 and 730nm). Panels consisted of antibodies conjugated to the following fluorophores: BV421, BV480, AF488, JOPRO-1, AF546, eF615, AF647, AF700. Images were tiled and merged using LAS X Navigator software. To ensure accurate representation and minimize selection bias at least 50% of the tissue was imaged or 5 follicles were captured on average in line with previously published research in our lab (24, 30). Replicate markers, shared among all panels, were also included in the analysis to ensure reproducibility across tissue sections and orientations. For analysis, we focused on well-defined areas devoid of background staining and quantified the data either as relative frequencies or as cell counts normalized to total tissue area (**Main Figures** and **Supplementary Figure 1A**). The latter is an alternative way to present the data that takes into consideration potential differences in tissue size allowing for this parameter to be controlled during quantification. Cell numbers (counts) for this alternative analysis were obtained from HistoCytometry using the program FlowJo whilst the total tissue area was calculated using the Imaris software through the surface creation module (**Supplementary Figure 1B**). The Imaris software was also used to calculate the number of FDC-bound virions in RNAscope analysis. Quantitation in this case was performed using the Spots creation feature after appropriately masking the vRNA+ channel to reveal colocalization with FDC surfaces. To minimize spectral spill-over among the various channels, fluorophore emission was collected on separate detectors with fluorophores being excited sequentially (2-3 fluorophores/sequential). Furthermore, spectral areas corresponding to each fluorophore were carefully selected after testing each fluorophore separately to verify the exact emission curve. Of note, staining of tissues with single antibody-fluorophore specificities was performed under the exact same conditions as the full panels in order for any protocol-associated shifts in emission curves to be accounted for. Fluorophore spillover, where present, was corrected by imaging tissues stained with single antibody-fluorophore combinations and by creating a compensation matrix *via* the Leica LAS-AF Channel Dye Separation module (Leica Microsystems) per user’s manual. Images were collected as z-stacks and are presented as maximum intensity projections (MIP) throughout the manuscript. Spillover corrected images were analyzed with the Imaris software version 9.6.0 (Bitplane Scientific) and the HistoCytometry pipeline was applied as previously described (29, 31). Briefly, 3- dimensional imaging datasets were segmented based on their nuclear staining signal and marker(s) of interest using the Surface Creation Module and average voxel intensities for all channels were calculated. Average voxel intensities for all channels of interest, within these surfaces, along with the X, Y positioning of the cell centroids (sphericity, volume), were then exported to Excel and combined into a unified spreadsheet file (comma separated values format). This spreadsheet was then imported into the FlowJo v 10 program for further analysis.

### Statistical Analysis

Statistical analyses were performed with GraphPad Prism 9.0 (GraphPad Software, Inc, San Diego, CA). We compared differences among groups using one-way analysis of variance (ANOVA) and Tukey’s multiple-comparison posttest. Differences between groups were considered to be significant at a *P* value of <0.05.

## Results

### Evaluation of GC Reactivity in Lymphoid Tissues

**Figure 1:**
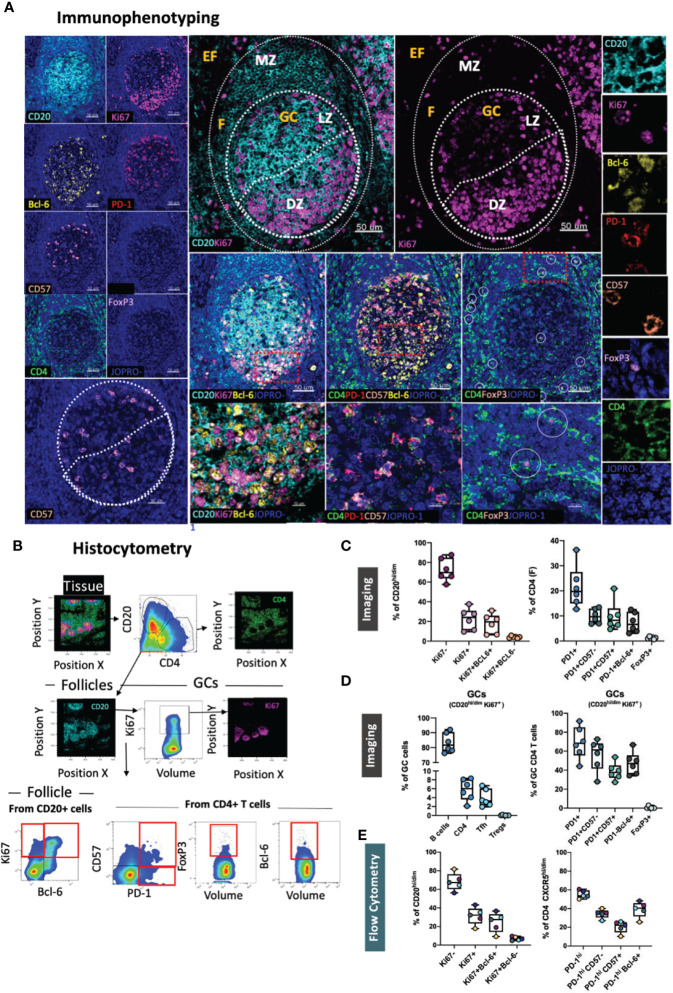
Development of a multispectral panel for the characterization of GC reactivity. **(A)** Representative example of Ki67 (magenta), CD20 (cyan), PD-1 (red), Bcl-6 (yellow), CD57 (orange), CD4 (green) and FoxP3 (pink) staining patterns in a tonsillar tissue section (left and right panels) and concomitant B-cell follicle and GC boundary delineations (Extrafollicular CD20lo and Follicular CD20hi/dim; Mantle Zone; MZ: CD20dimKi67lo, Light Zone; LZ: CD20hiKi67lo and Dark Zone; DZ: CD20dim Ki67hi) (middle panel). Circles in lower middle panels denote single FoxP3 events. **(B)** Histocytometry immunophenotyping gating strategy used for the sequential identification of B cells (CD20+Bcl-6+/- Ki67+/-), Tfh (CD4+PD-1hiBcl-6+/- CD57+/-), and Tfr (CD4+FoxP3+) in tonsillar tissue after nuclear staining based cell segmentation on Imaris and FlowJo analysis. F and EF and GC areas were defined based on the respective staining signals for CD20, Ki67 and CD4 and manually gated on FlowJo to extract frequencies for specific tissue localities. **(C)** Box plots showing the representative frequencies of B cell and CD4+ T-cell populations (Tfh, Treg) in B-cell follicles (CD20^hi/dim^ areas) as defined by the expression of Ki67/Bcl-6, PD-1/CD57/Bcl-6 and FoxP3 in tonsillar tissue after application of the GC reactivity pipeline. Each box plot circle represents an individual follicle. Colors represent different phenotypic groups. **(D)** Box plots showing the relative frequencies of B-cells, CD4 T cells, Tfh and Tregs (Tfr) as well as the frequencies of select CD4 T cell subpopulations in GCs (CD20^hi/dim^ Ki67+ areas) expressed as percentages of total GC cells measured. Circles represent distinct follicles and colors denote individual subpopulations. **(E)** Box plots showing the relative frequencies of B cell and CD4+ T-cell populations in B-cell follicles as measured by flow cytometry (n=5). CD4 T cells proximal to follicular localities and within GCs were identified through the expression of the B-cell follicle-specific chemokine receptor CXCR5 (CXCR5^hi/dim^). Images were acquired at 40x (NA 1.3) with 1% magnification. Scale bars are 50um, 30um (CD57 positional image) and 10um (lower panel zoomed details) respectively.

To investigate GC reactivity, we designed a panel combining the GC relevant markers CD20, Ki67, Bcl-6, PD-1, CD4, CD57 and FoxP3 (**Figure 1A**) and performed quantification of immune cell frequencies in selected tonsillar topologies by HistoCytometry (29). We used CD20, Ki67 and Bcl-6 to map B cell follicles (CD20^hi/dim^) or GCs (CD20^hi/dim^Ki67^+^) as well as for the delineation of the mantle zone (MZ; CD20^dim^Ki67^-^) where most naïve B-cells reside, dark zone (DZ; CD20^dim^Ki67^+^), where clonal expansion and antigen receptor diversification occurs, and light zone (LZ; CD20^hi/dim^Ki67^+/-^) where B-cells undergo selection (19). Bcl-6 is a transcriptional repressor that is required for mature B-cell GC formation. Within the B-cell lineage, Bcl-6 expression is mainly confined to GC B cells where it acts to promote the selection of B-cells by silencing the antiapoptotic molecule Bcl-2, enhancing the ability of GC B cells to tolerate DNA damage, preserve B cell identity and fine-tune BCR mediated responsiveness (19) Bcl-6 is also expressed in CD4+ T-cells and has been shown to direct Tfh lineage commitment (32). Bcl-6 further distinguishes i) follicular B-cell subsets; GC B-cells (DZ: CD20^dim^Ki67^+^Bcl-6^+^, LZ: CD20^hi/dim^Ki67^+/-^Bcl-6^+^) and non-GC (CD20^hi^ Ki67^-^Bcl-6^-^) and ii) Tfh cell subsets (PD-1^hi^ Bcl-6^hi/dim^) (**Figure 1B**). Furthermore, we used the marker CD57, an epitope expressed in a subset of CD4 T cells that are positive for CD69 and CD45RO and detected in approximately 15-25% of tonsil CXCR5+ CD4 T cells (33), to track two distinct GC-Tfh populations (CD57^+^ and CD57^-^) that have been shown to possess divergent immunophenotypes (24). Potential regulatory CD4 T cells were also measured and localized based on the expression of the transcription factor FoxP3. Quantitative analysis revealed an overall higher frequency of Ki67^-^ cells within follicular areas (**Figure 1C**). Ki67^+^ cells were in their majority Bcl-6^hi^ and represented 10-38% of the total CD20^hi/dim^ population. Bcl-6^lo^ B-cells were also present within the Ki67^+^ DZ compartment but at a very low frequency (<1%) (**Figure 1C**) Our analysis thus, confirms Ki67 and Bcl-6 co-staining as a meaningful way to topologically define the LZ/DZ compartments. When CD4 T-cells were examined, a variable percentage of PD-1^hi^ CD4+ T-cells (Tfh) was observed among the follicles measured. Within follicular areas, PD-1^hi^ cells represented on average 20% of the total CD4 population (**Figure 1C**) or 69% of the total CD4 population when a stricter follicular definition was applied that excluded PD-1^lo^ and PD-1^dim^ CD4 T -cells residing outside the LZ/DZ localities (GC only) (**Figure 1D** and **Supplementary Figure 1C**). Analysis of Tfh subpopulations revealed a trend for higher frequencies of CD57^-^ Tfh within tonsillar B-cell follicles compared to CD57^+^ irrespective of the B-cell follicle definition used with the latter being localized closer to the DZ as we have previously reported (24) (**Figures 1A, C, D**). We also measured Bcl-6 and FoxP3 expression within follicular areas. We found 5-10% of follicular CD4 T-cells to possess the Bcl-6^hi^ signature, whilst FoxP3+ expression was seen in <2% of CD4 T-cells (**Figure 1C**). In GCs, B-cells were the most abundant population (~83% of GC cells) whilst CD4 T cells, Tfh and Tfrs represented ~6%, 4% and 0,03% of the total cells respectively as measured by HistoCytometry (**Figure 1D**). Consistent with previous reports, FoxP3 expression within tonsillar follicular areas was detected primarily at the B-T cell border and only rarely in the GC (34) (**Figure 1A**). Cross-validation of the studied populations by flow cytometry revealed comparable frequencies and trends (**Figure 1E** and **Supplementary Figures 1C, D**). Our GC reactivity pipeline thus tracks B-cells, CD4+ T-cells (including Tfh) and Tregs in tonsillar tissue subanatomical localities accurately and in line with previously published reports.

### Assessment of Tissue-Specific T-Cell Regulation

**Figure 2:**
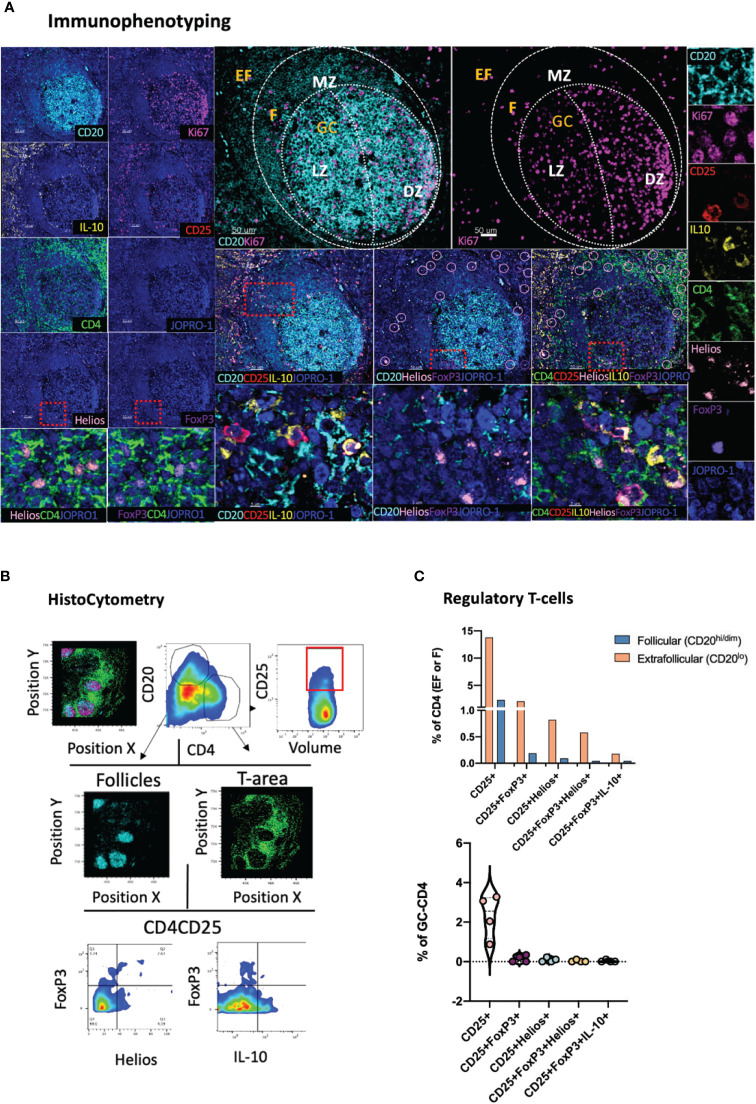
Development of a multispectral panel for the assessment of tissue-specific T-cell regulation. **(A)** Representative example of antibody staining patterns for Helios (pink), CD20 (cyan), CD25 (red), IL-10 (yellow), Ki67 (magenta), CD4 (green) and FoxP3 (purple) in a tonsillar tissue section (left and right panels) and distribution of each marker with respect to the MZ, LZ and DZ (middle panels). Circles (pink/purple) in lower middle panels denote individual Helios (pink) and FoxP3 (purple) events. **(B)** Immunophenotyping gating strategy used for the identification of Tregs and Tfrs in lymphoid tissues of interest based on the expression and coexpression of the transcription factors FoxP3, Helios, the protein marker CD25 and IL10 in histocytometry. **(C)** Bar graphs (upper row) and violin plots (lower row) showing the frequencies of Tregs and Tfrs in follicular and extrafollicular areas and frequencies of Tfr subsets in a tonsillar tissue section. Circles in violin plots represent individual follicles whereas colors represent different phenotypic subgroups. Images were acquired at 40x (NA 1.3) with 1% magnification. Scale bars are 50um, 10um (zoomed in details) and 5um (Helios and FoxP3 staining patterns) respectively.

FoxP3+ CD4 T-cells have been shown to represent critical regulators of the GC size and activity, including that of Tfh cells, in both mice (35) and humans (36). To allow for a more detailed study of regulatory T-cells in lymphoid tissues, we designed a dedicated panel consisting of the Treg- specific markers CD4, CD25 and FoxP3 all of which are typical phenotypic markers of natural (thymus-derived) Tregs (37) (**Figure 2A**). Helios, an Ikaros transcription factor family member that has been shown to stabilize the Treg phenotype (38), was also included in the panel as it has been associated with a better capacity for immunosuppression in some studies (39) as well as with vaccine-induced Treg kinetics in tonsillar tissues (30). We also probed for IL-10, a cytokine linked to the functional capacity of Tregs, in line with reports suggesting a role for Tfr cell-derived IL-10 in the modulation of GC responses in acute viral infection (40, 41). In line with previous reports, quantitative analysis of relevant Treg populations in tonsillar tissue using the HistoCytometry pipeline revealed a higher percentage of regulatory T cells in EF areas (CD20^lo^) compared with follicular areas (30, 34) (**Figures 2B, C**, upper graph). This trend was true for all populations measured irrespective of their specific phenotype (CD25^hi^FoxP3^+^ or CD25^hi^Helios^+^ or CD25^hi^FoxP3^+^Helios^+^ or CD25^hi^FoxP^+^IL10^hi^). Within GCs, regulatory T-cells (Tfr) were rare. The frequency of CD25^hi^FoxP3^+^ Tfrs in all follicles combined did not exceed 0.2% whilst that of CD25^hi^FoxP3^+^Helios^+^ and CD25^hi^FoxP3^-^Helios^+^ was lower than 0.05% (**Figure 2C**, lower graph). CD25+FoxP3^+^ Tregs had the highest frequency in the T cell zone (up to 2% of total CD4+ T-cells). We also observed the presence of CD25^hi^FoxP3^+i^IL10^hi^ Tregs, albeit at a low frequency (~0.3% of EF CD4 T-cells). Taken together, these data suggest that CD25, FoxP3, Helios and IL10 in combination with CD4, CD20 and Ki67 can be used to map and quantify Tregs and Tfrs effectively in microanatomical sites of interest including the GC, B cell follicle – T cell zone border where Tfr-mediated suppression may be more efficient (34) as well as in paracortical T-cell zones (CD20^-^CD4^+^ areas).

### Topological Quantification of B-Cell Immunity

**Figure 3:**
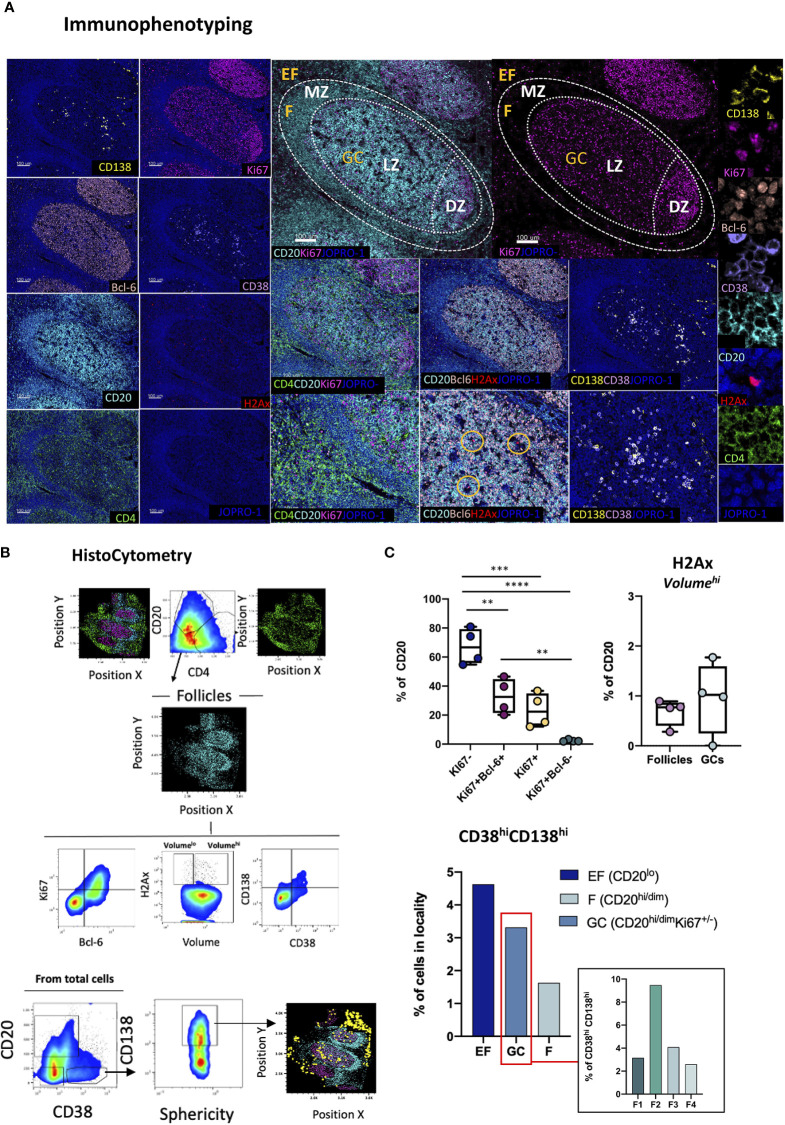
Development of a multispectral panel for the topological examination of B cells. **(A)** Representative example of antibody staining patterns for CD138 (yellow), Ki67(magenta), Bcl-6 (orange), CD38 (violet), CD20 (cyan), H2Ax (red), CD4 (green) and nuclar staining (JOPRO-1, blue) and distribution of each marker with respect to the MZ, LZ and DZ (middle panels). Orange circles in the lower row images denote H2Ax events. **(B)** Gating strategy used for the immunophenotyping of various B cell populations and CD38hiCD138hi cells in GC (CD20^dim^Ki67^hi^), F (CD20^hii/dim^) and EF (CD20^lo^) areas. **(C)** Box plots showing the frequencies of various B cell populations (as defined by the expression of Ki67 and Bcl-6), H2Ax Volume^hi^ CD20+ cells and CD38^hi^CD138^hi^ cells in a representative tonsil tissue section. Each circle represents a distinct follicle and each color denotes a distinct subpopulation. p values were derived from ANOVA analysis (Tukey’s multiple comparisons test). Images were acquired at 40x (NA 1.3) with 1% magnification. Scale bars are 100um for all panels displaying an entire follicle), 50um for the middle panel close ups and 5um for the right panel single staining close ups. **p < 0.01, ***p < 0.001, ****p < 0.0001.

Whilst a topological examination of B cells in GCs can be achieved through the combinatorial examination of CD20, Ki67 and Bcl-6, a more granular view of B cell subsets is often necessary to appreciate the microenvironment that leads to the development of GC B cells and plasma cells. A deeper understanding of the role that specific B cell subsets might have in modulating the evolution of GC reactions is also desirable (42). This topological distribution becomes of particular relevance in the context of infection, such as chronic HIV infection, where B-cell responses become overtly dysregulated (43). To address the limitations of a tripartite CD20-Ki67-Bcl-6 analysis, a panel incorporating three additional markers, namely CD138, CD38 and pH2Ax was developed (**Figure 3A**). In this panel CD38, a marker of activated T and B lymphocytes (44) is used alongside the plasma cell marker CD138 (45) to inform on the tissue-specific positioning of B cells (CD20^hi^CD38^lo^; CD20^hi^ CD38^dim^ CD138^lo^) and plasma cells (CD20^lo^ CD38^hi^ CD138^hi^) (**Figure 3B**) in follicular and extrafollicular (CD20-CD4^hi^) areas. In addition, pH2Ax, a histone that becomes rapidly phosphorylated in response to DNA double-strand breaks (DSBs) (46) serves as proxy for class switch recombination (CSR) (47). First, B-cells in tonsillar tissue were dissected by means of Ki67 and Bcl-6 expression. As expected, and consistent to our previous analysis (**Figure 1C**), we found a significantly higher frequency of Ki67^-^ B cells within total follicular areas compared to the frequencies of all other measured populations (*vs* Ki67^+^, p=0.004; *vs* Ki67^+^Bcl-6^-,^ p <0.0001 and *vs* Ki67^+^Bcl-6^+^; p=0.0028) (**Figure 3C**, upper panel). Within B cell follicles, we also measured the frequency of pH2Ax positive CD20+ cells. Since phosphorylation of H2AX can take place in cells undergoing cell death as well, we focused our analysis on cells that displayed higher overall volumes in HistoCytometry as measured by nuclear staining (Volume^hi^ for live cells). We found that pH2Ax/Volume^hi^ frequencies were higher in GCs as compared to the total follicle, consistent with the role of GCs in class switch recombination and somatic hypermutation (**Figures 3B** for gating and **3C**, upper right panel). To address the low overall frequency of pH2Ax (Volume^hi^) events in tonsils, a confirmatory analysis of the specificity of the detected signal by flow cytometry was undertaken. Unswitched (IgG^lo^) B-cells within distinct tonsillar localities were defined by the combined expression of CD20 and CD38 as CD20^dim^CD38^lo^, CD20^hi^CD38^dim^ and CD20^lo^CD38^hi^. We observed pH2Ax frequencies to be the highest within the CD20^hi^CD38^dim^ compartment (**Supplementary Figure 2A**). pH2Ax expression was also examined in LN sections from HIV+ individuals. The latter analysis revealed higher frequencies of pH2Ax (Volume^hi^) in the follicles and GCs of HIV+ LN specimens compared to the frequencies seen in HIV- tonsillar tissues, consistent with the higher levels of GC reactivity expected in chronic HIV+ infection (**Supplementary Figure 2A**). Taken together these data suggested that pH2Ax is meaningful biomarker for monitoring B-cell responses. We also addressed in our analyses the frequency of CD38^hi^CD138^hi^ cells in tonsillar EF, F and GC bound localities and observed a higher prevalence CD38^hi^CD138^hi^ cells in EF areas (~5% of total EF cells) and GCs as compared to CD20^hi/dim^ areas, which confirmed the MZ as the area with the higher density of naïve B cells. When different follicles were examined, variable frequencies of CD38^hi^CD138^hi^ cells were found (0.65-3% of total cells in CD20^hi/dim^ localities) (**Figure 3C**, lower panel). Our data therefore confirm the combination of these six B cell specific markers as a valid means to interrogate the topologies, and associated frequencies of several relevant B cell subsets in lymphoid tissues for analyses of B cell dynamics.

### Evaluation of CD8 T-Cell Positioning and Function

**Figure 4:**
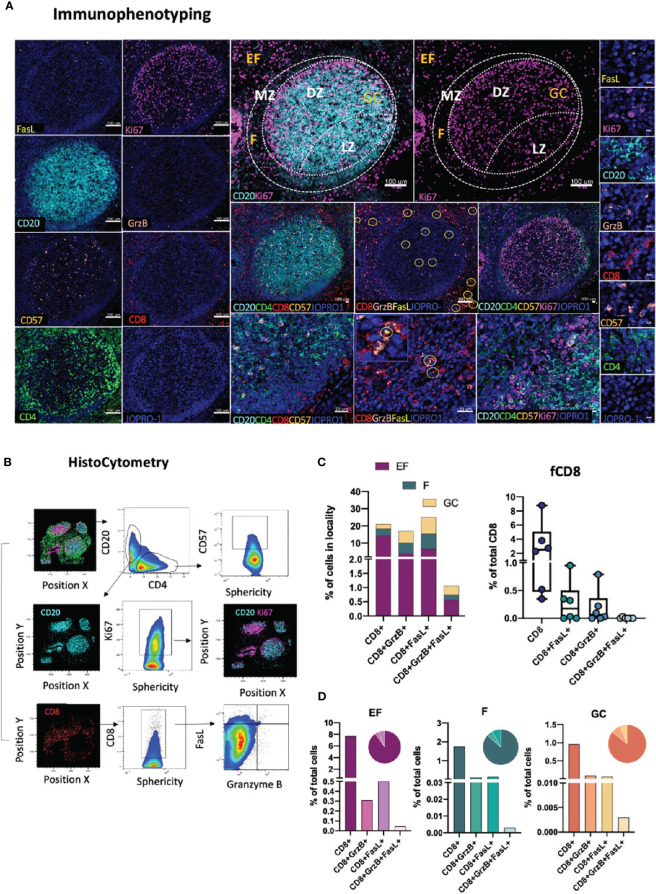
Development of a multispectral panel for the topological examination of CD8 T cells. **(A)** Representative example of antibody staining patterns for FasL (yellow), Ki67 (magenta), CD20 (cyan), Granzyme B (pink), CD57 (orange), CD4 (green) and the nuclear marker JOPRO-1 (blue) and distribution of each protein marker with respect to the MZ, LZ and DZ (middle panels). Yellow circles in lower middle panels denote individual FasL events. **(B)** Gating strategy used for the immunophenotyping of various CD8 T cell populations (CD8^hi^; CD8^hi^GrzB+, CD8^hi^FasL+ and CD8^hi^ GrzB+ FasL+) in in GC (CD20^dim^Ki67^hi^), F (CD20^hi/dim^)and EF (CD20^lo^) areas. **(C)** Box plots and bar graphs showing the relative frequencies of four distinct CD8 T cell populations in EF, F and GC; the frequencies of follicular CD8^hi^; CD8^hi^GrzB+, CD8^hi^FasL+ and CD8^hi^GrzB+ FasL+ in six distinct follicles and **(D)** proportion of each CD8+ cell subset within each locality (EF, F and GC) as a percentage of total cells. Each circle represents a follicle and each color stands for a distinct subpopulation. Images were acquired at 40x (NA 1.3) with 1% magnification. Scale bars are 100um for all panels displaying an entire follicle), 20um in the middle panel close ups and 5um in the right panel single staining close ups.

Another tissue-resident immune subset of great importance, especially in the context of viral infection, is CD8 T-cells. Within tonsillar tissues, CD8 T cells have been shown to localize in extrafollicular (CXCR5^-^CD8^+^) and to a lesser extend within follicular (CXCR5^+^CD8^+^) spaces, with the latter (follicular CD8+ T-cells or fCD8) displaying an early effector phenotype associated with a non-cytolytic (granzyme A^+^perforin^-^) profile that sets them apart from their CXCR5- counterparts (48). Traditionally, cytotoxic T cells, such as CD8 T cells, have been defined by the expression of perforin, granzyme B as well as their expression of high levels of the transcription factor T-bet that regulates the cytotoxic effector gene program (49) In the context of lymphoid tissues however, and in particular in HIV/SIV infection, CD8 T cells have been shown to possess a low level, discordant expression of perforin and granzyme B (50). To address the topology of CD8 CTLs in lymphoid tissues we designed a panel using the lineage-specific marker CD8 (CD8^bright^) in combination with Granzyme B, FasL, an alternative major mediator of CTL killing, and CD57, a marker of senescence of CD8 T cells (51), as well as the GC and T-cell zone markers CD20, Ki67 and CD4 (**Figure 4A**). These markers allow for the spatial distribution of CD8 populations to be dissected by means of marker co-expression as CD8^bright^GrzB^+/-^, CD8^bright^FasL^+/-^ or CD8^bright^GrzB^+^FasL^+^ either in stand-alone topologies or in relation to the positioning of intrafollicular CD4^+^CD57^+/-^ as well as CD20^hi/dim^Ki67^+/-^ B-cells (**Figures 4A, B**). In absence of the lineage marker CD3, analysis in of CD8 T cells in tonsillar tissues was restricted on CD8^bright^ T cells which excluded the majority of CD8^dim^ CD3^-^ T cells present in the tonsils (**Supplementary Figure 2B**). However, in cases where NK cell frequencies are of interest, CD3 can be easily incorporated into the panel in the place of CD57 to facilitate the characterization of the CD3^-^CD8^dim^ population (**Supplementary Figure 2C**). In the absence of perforin staining, we use granzyme B and FasL – two molecules with potential for upregulation in infection, in particular HIV infection (31, 52) to denote CD8 T cells with potential for cytotoxicity. As expected, analysis of CD8 distributions in tonsillar tissue with histocytometry revealed a higher frequency of CD8 T cells in EF localities than in F or GC localities and comparable frequencies of CD8 within the latter two (**Figure 4C**, upper panel). We also found considerable variations in the frequencies of follicular CD8 T-cells amongst different follicles that ranged from 0.3 to 9% of total CD8 T-cells. In their majority fCD8 lacked expression of Granzyme B+ or FasL+ with only a small minority expressing either of these two markers (<0.6% of total GC cells). Double positive GranzymeB^+^ FasL^+^ fCD8^+^ T-cells were also rare (<0.020% of total GC cells) in tonsils (**Figure 4C**, lower panel) but their expression is upregulated in HIV infected LNs (**Supplementary Figure 3A**). Despite the differences however in the CD8+ T cell subset frequencies found in tonsils, no significant differences were observed when the relative proportions of each population within EF, F and GC areas were examined (**Figure 4D**). Taken together, our data show that in line with previous reports, CD8+ T-cells in tonsillar tissues are mainly extrafollicular in nature and display a phenotype that is consistent with a reduced cytotoxic potential. HIV infection however can increase the frequencies of these rare populations, including those of CD8+ FasL+ T-cells in follicular localities (**Supplementary Figure 3A**). Application of this panel thus can provide important information on CD8 T-cell GC infiltration and fCD8 dynamics in pathological contexts. It can also be used to reveal possible functional correlations between CD8-CD4 T cells and CD8-B cells especially in HIV infection where Tfh have been shown to represent an active tissue reservoir (27).

### Topological Distribution of Monocytic and Inflammatory Markers

**Figure 5:**
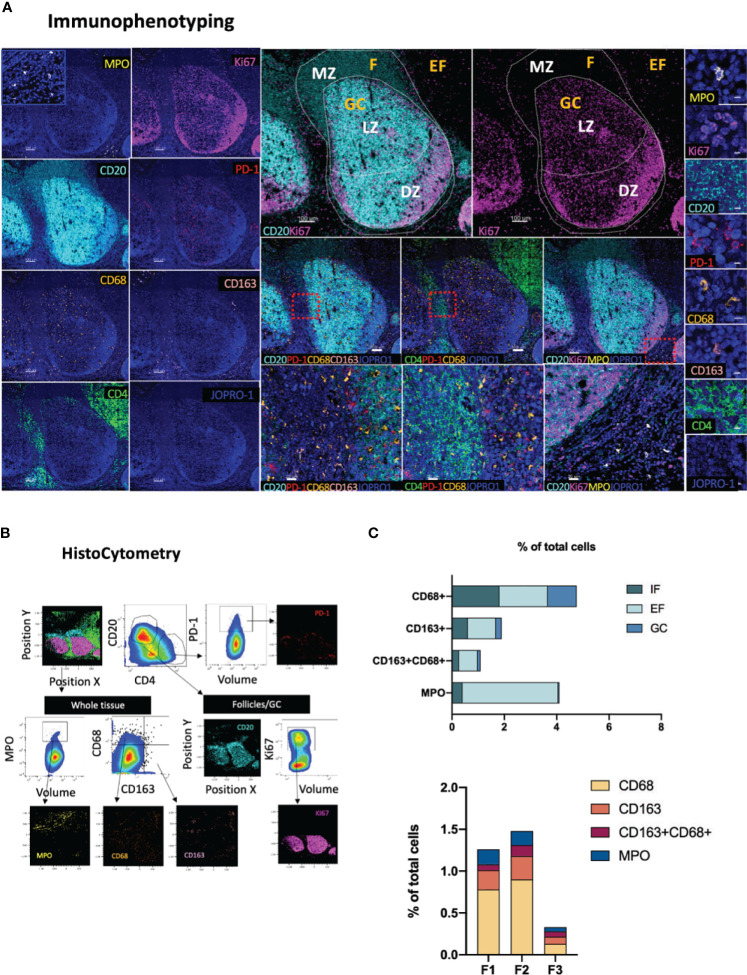
Development of a multispectral panel for the topological examination of monocytes and inflammatory markers. **(A)** Representative example of antibody staining patterns for MPO (yellow), CD20 (cyan), Ki67 (magenta), PD-1 (red), CD68 (orange), CD163 (pink), CD4 (green) and JOPRO (nucleus, blue) and distribution of each protein marker with respect to the MZ, LZ and DZ (middle panels). Red dotted enclosures in lower middle panels denote the areas for which zoom ins are given (lower middle panels). **(B)** Gating strategy used for the immunophenotyping of neutrophils (MPOhi), monocytic populations (CD163^hi^CD68^lo^, CD68^hi^CD163^lo^, CD68^hi^CD163^hi^) and Tfh (CD4+PD-1^hi^) in GC (CD20^dim^Ki67^hi^), F (CD20^hi/dim^) and EF (CD20^lo^) areas. **(C)** Bar graphs showing the relative frequencies of MPO^hi^ and various monocytic populations in intrafollicular (IF), EF and GCs as well as the frequencies of the same populations in three distinct follicles imaged. Images were acquired at 40x (NA 1.3) with 1% magnification. Scale bars are 100um for all panels displaying an entire follicle), 20um in the middle panel close ups and 5um in the right panel single staining close ups.

Antigenic challenge upregulates inflammatory markers in draining lymphoid sites and encourages innate immune cell infiltration (53). Whilst in a non-pathological context inflammatory processes serve to orchestrate innate and adaptive immune responses (54), chronic inflammation, such as the one triggered by HIV infection, can profoundly dysregulate immune dynamics through the disruption of immune cell trafficking (55), induction of skewed immune cell topologies (31), and through dysregulation of immune cell ratios (56). The spatial dynamics of immune cells with pro- or anti- inflammatory potential therefore holds particular prognostic relevance in studies of infection and vaccination. Towards this end, we developed a panel consisting of the markers CD163, CD68, MPO, CD4, CD20, Ki67 and PD-1 (**Figure 5A**). In this panel, subsets of the monocyte/macrophage lineage are defined as CD163^hi^CD68^lo^, CD163^lo^CD68^hi^ and CD163^hi^CD68^hi^ based on the expression of the markers CD163 (a type B scavenger receptor) and CD68, a marker expressed by mature macrophages and resident histiocytes (57, 58). In addition, cells expressing myeloperoxidase (MPO^hi^), a heme-containing enzyme found in the primary azurophilic granules of neutrophils (59), one of the main cell types involved in the inflammatory response- (60) are mapped relative to GC or Tfh positioning (CD4^+^PD-1^+^) (**Figure 5B**). Application of this panel to tonsillar tissue revealed that MPO^hi^, CD163^hi^CD68^lo^ and CD163^hi^CD68^hi^ cells are preferentially localized in EF areas with only exception being CD163^lo^CD68^hi^ cells which showed comparable distributions in EF (CD20^lo^) and F areas (1.85% and 1.81% of total cells in EF and F areas respectively) consistent with previously published reports (61) (**Figure 5C**). In GCs, CD163^lo^CD68^hi^ cells were the most abundant of the four populations measured (1.1% of total cells), a signal most likely originating from tingible body macrophages (TBMs) (62). No significant proportional variations were observed in the frequencies of these four subsets among individual follicles (**Figure 5C**, lower graph). Taken together, our data suggest that meaningful information on the position and relative abundance of pro-inflammatory cells in lymphoid tissues can be obtained by co-staining for MPO, CD163 and CD68.

### Evaluation of GC Microarchitecture

**Figure 6:**
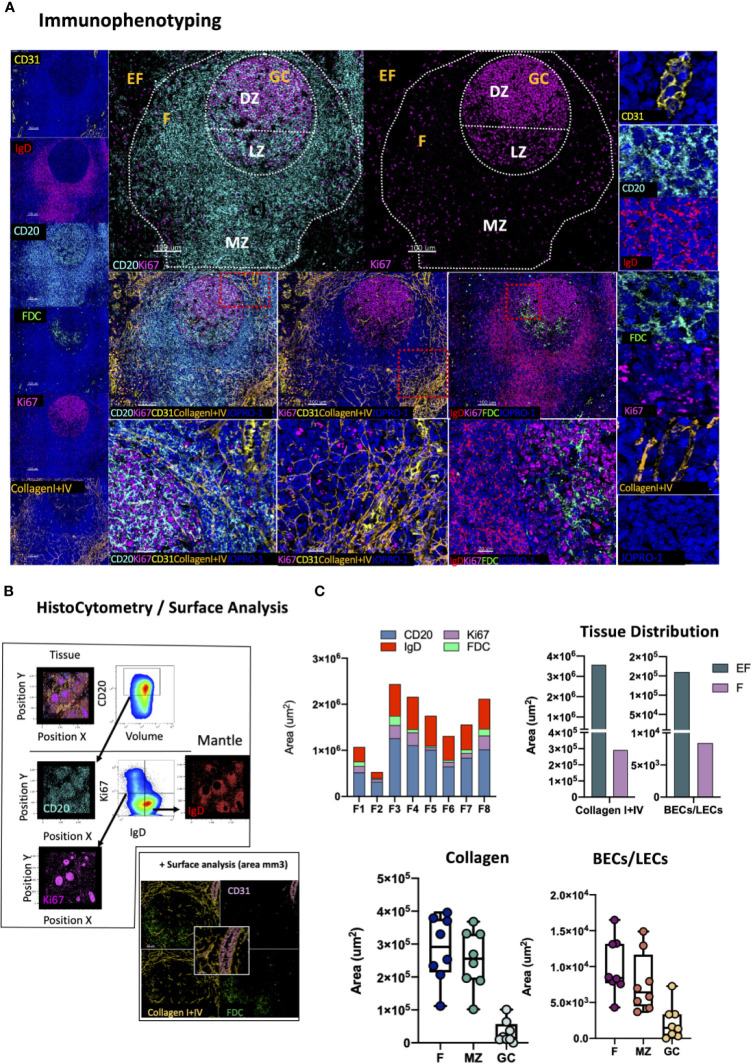
Development of a multispectral panel for the evaluation of GC microarchitecture. **(A)** Representative images showing staining for CD31 (yellow), IgD (red), CD20 (cyan), FDC (green), KI67 (magenta) and Collagen I+IV (orange) in a tonsillar tissue section and individual protein marker distributions with respect to the MZ, LZ and DZ of a B-cell follicle (middle panels). Red dotted square enclosures in lower middle panels denote the areas that are presented magnified in the lower middle panels. **(B)** Gating strategy used for the sequential positional immunophenotyping of naïve B-cells (IgD^hi^) in F (CD20^hi/dim^) areas using histocytometry. **(C)** Box plots showing the areas (um3) occupied by each individual microarchitectural component namely B-cell follicle; CD20^hi/dim;^ mantle zone: IgD^hi^, GC (CD20^dim^Ki67^hi^) and FDC network (FDC^hi^) in eight distinct B cell follicles as measured by surface analysis in the program Imaris as well as the relative areas occupied by Collagen (Collagen I+IVhi) and vascular and lymphatic endothelium (CD31^hi^CollagenI+IV^hi^) in EF areas, F areas and GCs. Images were acquired at 40x (NA 1.3) with 1% magnification. Scale bars are 100um for all panels displaying an entire follicle), 20um in the middle panel close ups and 5um in the right panel single staining close ups. BEC, Blood Endothelial Cell; LEC, Lymphatic Endothelial Cell.

The preservation of lymphoid tissue architecture, and by extension GC microarchitecture is of paramount importance for the evolution local immune responses. Loss of GC topology due to ageing or pathology negatively impacts responses to infection and vaccination (63, 64). Reversible lymphatic remodeling occurs with every round of antigenic challenge and is mediated by distinct stromal cell populations with differential expression of CD31 (PECAM1) including T cell zone reticular cells (TRECs), follicular dendritic cells (FDC), marginal reticular cells (MRC), lymphatic endothelial cells (LECs), blood endothelial cells (BEC) and high endothelial cells (HECs) (65, 66). HIV infection in particular profoundly perturbs the architecture of lymphoid organs through progressive collagen deposition and fibrosis, that disrupts and damages the important follicular reticular cell (FRC) network (13, 67) To map stroma and GC specific changes we developed a panel consisting of markers CD31, IgD, CD20, FDC, Ki67, Collagen I and Collagen IV (**Figure 6A**). In this panel, CD20, Ki67 and IgD are used to define the follicular mantle (CD20^dim^IgD^+^), GC-LZ (IgD^-^CD20^hi^Ki67^lo^) and GC-DZ (IgD^-^CD20^dim^Ki67^hi^) for assessment of GC reactivity as well as quantification of naïve CD20^dim^IgD^hi^ or non-GC memory (CD20^dim^IgD^lo^ Ki67^-^) B cells (**Figure 6B**). FDCs hold a key role in the maintenance of GCs and retention of antigen (66) and their disruption has been shown to directly impact GC stability (68), especially in the context of chronic HIV infection (69, 70). FDC disruption can be measured by applying this panel as a reduction of the total area occupied by these cells compared to the total area of the follicle as previously reported (71). Topological mapping of BECs and LECs is also possible through combinatorial analysis of CD31 and Collagen I+IV staining (CD31^hi^Collagen^hi^) whilst cumulative changes in the reticulum can be traced by quantification of the total collagen^hi^ signal present in select topological compartments (**Figure 6B**). We applied this panel to map intrafollicular variation in tonsillar tissues and found considerable variation amongst follicles in CD20^hi/dim^, CD20^dim^Ki67^hi^, IgD^hi^ and FDC^hi^ areas, which was reflective of the overall size of individual follicles (**Figure 6C**, first graph). In addition, analysis of collagen I+IV and CD31 staining distribution either as a single stain (Collagen I+IV^hi^) or in combination (vascular and lymphatic endothelium) showed preferential segregation of BECs and LECs in extrafollicular spaces and within MZs but only minimal staining was seen in GCs (**Figure 6C**).Taken together these data suggest lymphoid tissue specific microarchitectural niches can be successfully mapped by CD20, IgD, Ki67, FDC, CD31 and collagen staining to yield important information regarding stromal changes, especially in the context of pathology when variations are likely to be tied to clinical outcomes.

### Positioning of Tissue-Specific DC Subpopulations

**Figure 7:**
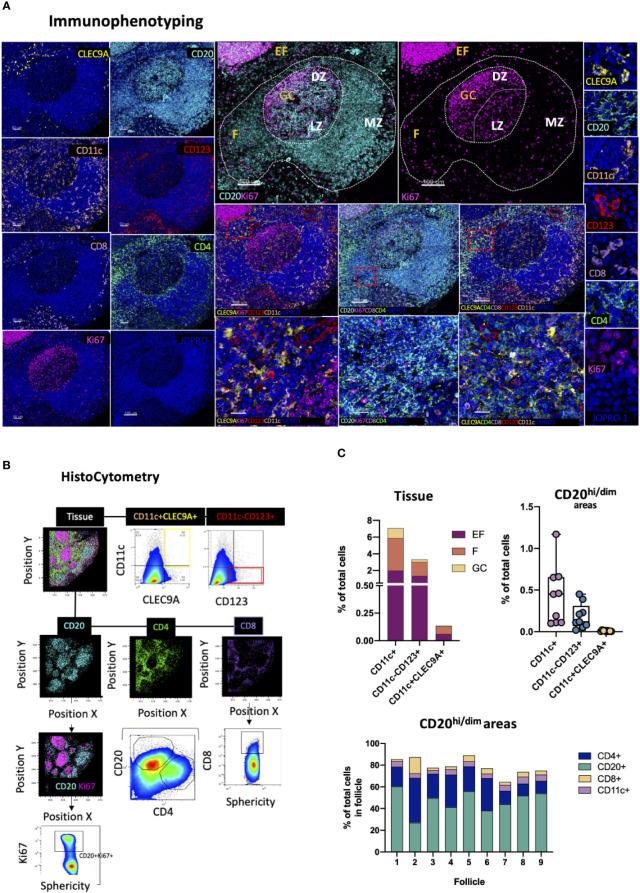
Development of a multispectral panel for the topological examination of DCs. **(A)** Representative images showing staining for CLEC9A (yellow), CD20 (cyan), CD11c (orange), CD123 (red), CD8 (pink), CD4 (green), Ki67 (magenta) and JOPRO-1 (blue) in a tonsillar tissue section and the distribution of each protein marker with respect to the MZ, LZ and DZ (middle panels). Red dotted square enclosures in lower middle panels denote the areas that are presented magnified in the lower middle panels. **(B)** Gating strategy used for the sequential positional immunophenotyping of CD4, CD8, CD20, CD11c+, CD11c+CLEC9A+CD123- and CD11c-CD123+CLEC9A- in GC (CD20^dim^Ki67^hi^), F (CD20^hi/dim^) and EF (CD20^lo^) areas using histocytometry. **(C)** Box graphs and box plots showing the relative frequencies of CD11c+, CD11c+CLEC9A+CD123- and CD11c-CD123+CLEC9A- immune cells in tonsillar tissue as well as in CD20^hi/dim^ areas (upper panels) and relative frequencies of CD20, CD4, CD8 and CD11c+ immune cells in nine individual follicles. Images were acquired at 40x (NA 1.3) with 1% magnification. Scale bars are 100um for all panels displaying an entire follicle), 20um in the middle panel close ups and 5um in the right panel single staining close ups.

DCs are innate immune, antigen presenting cells with a key role in the induction of adaptive immune responses. Several DC subsets exist in humans with varying migratory potentials, tissue distributions, microanatomical compartmentalization and functions. These subsets fall under three major groups: CD11c+ myeloid DCs (mDCs), monocyte-derived DCs (moDCs) and CD123+ plasmacytoid DCs (pDCs) (72). The latter is a major interferon type I producing cell type in response to viral infection (73) and key spatiotemporal CD8 T-cell priming orchestrator within lymphoid tissues (74). CD141+ CLEC9A+ DCs of the CD11c+ subset on the other hand have been shown to be particularly efficiently in promoting naïve T cell activation (72). To map DC subpopulations in lymphoid tissues in studies of infection or vaccination we developed a panel consisting of the markers CD123, CLECL9A and CD11c in combination with CD20, Ki67 as well as CD4 and CD8 (**Figure 7A**). We used CD123, the a chain of the IL-3 receptor, for topological examination of pDCs (CD11c^-^CD123^+^CLEC9A^-^) (73) and CD11c, a β2-integrin (75), and CLEC9A, a C-type Lectin-like receptor (76) to interrogate the spatial distribution of CD11c^+^CD123^-^CLEC9A^+^ DC in subcapsular areas and conduits surrounding B cell follicles (**Figure 7B**). Cells of myeloid lineage (CD11c^hi^), CD8 T -cells and CD4 T -cells were also mapped using this panel to infer mechanistic insights regarding potential cell-to-cell interactions as well as for quantification of immune cell dynamics for hypothesis-driven discovery and experimental testing. Tonsillar tissue analysis revealed similar distributions of CD11c+, CD11c-CD123+ and CD11c+CLEC9A+ events between EF and perifollicular (CD20^hi/dim^, non-GC) spaces albeit at markedly different frequencies (**Figure 7C**, upper row, left). CD11c-CD123+ and CD11c+ CLEC9A+ events were rare within GCs (<0.3%) whilst CD11c+ cells were the most abundant of the three populations measured (~1% of total GC cells and 2% of total B-cell follicle cells) with individual distributions ranging from 0.1 to 1.2% within follicles (**Figure 7C**, upper low, right and lower panel). Some CD123+ staining was also observed in vascular lumen. This however was excluded from analysis at the stage of segmentation as it did not represent signal bound to a nucleus. Our data thus confirm that in tonsillar tissues CD11c-CD123+ and CD11c+CLEC9A+ signal is mainly located outside GCs which is consistent with the known positioning of DC and associated role of these cells in immune cell priming (53, 77).

### Detection of Cells Harboring Actively Transcribing HIV

**Figure 8:**
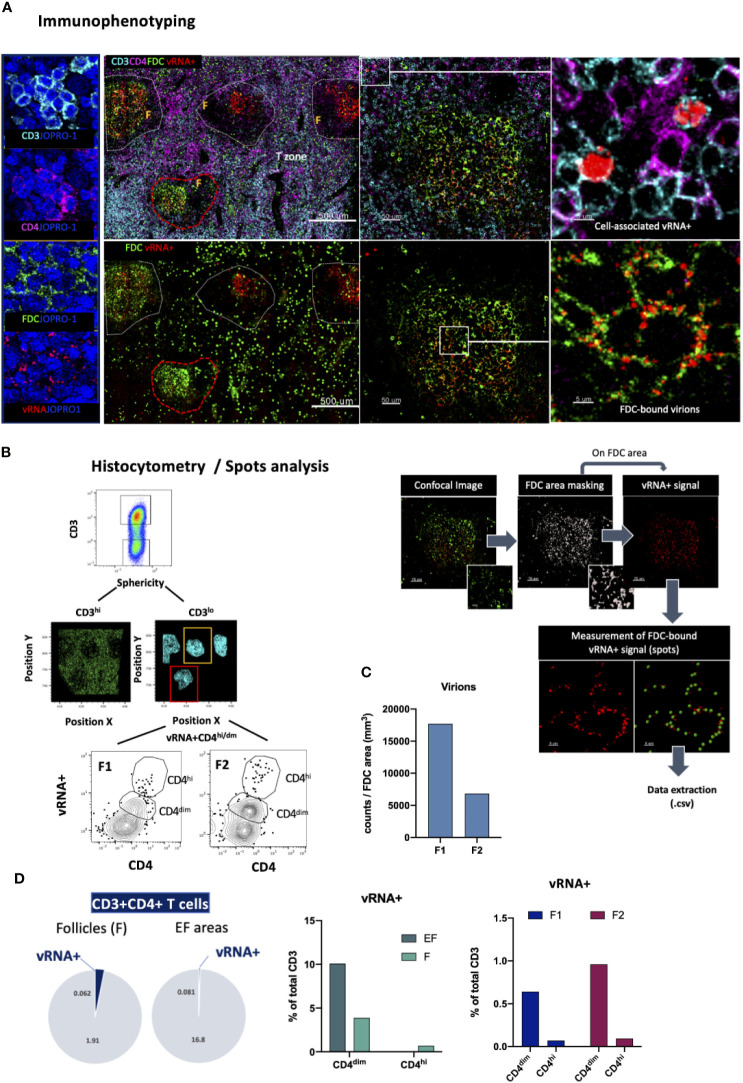
Immunofluorescent *in situ* hybridization (IF-FISH) for the visualization of actively transcribed HIV (vRNA+) in lymph nodes. **(A)** Representative images showing staining for CD3 (cyan), CD4 (magenta), FDC (green) and HIV RNA (red) and the nuclear stain JOPRO-1 (blue) in a lymph node section from a viremic HIV+ individual. Images shown (50um,5um) are sequential close ups of the red dotted enclosures and white enclosures respectively and depict the staining seen in a B-cell follicle and distinct patterns of associated vRNA+ staining (cell-associated vs FDC-bound virions). **(B)** Gating strategy used for the quantification of vRNA+ signal in CD3+ CD4^hi/dim^ T cells in two well defined B-cell follicles using HistoCytometry (left) and graphic panel summarizing the approach used to quantitate FDC-bound virions in lymphoid tissues (right). **(C)** Bar graph showing the FDC-associated virion burden in the two follicles examined as measured by histocytometry and surface spot analysis on Imaris. **(D)** Bar graphs showing the distribution of the vRNA+ signal in EF and F spaces as well as the relative distribution of the signal in CD4^hi^ *vs* CD4^dim^ cells and pie charts depicting the proportions of CD3+CD4+ T cells that are vRNA+/vRNA- in F and EF spaces as measured by HistoCytometry. The percentages given represent the frequency of each respective CD4 T cell population (EF vRNA+ or vRNA-/F vRNA+ or vRNA-) as a percentage of total cells d) and FDC-associated virion burden as Images were acquired at 40x (NA 1.3) with 1% magnification.

The determination of the localization of a pathogen following infection is of great importance for the development of successful, targeted prevention and cure strategies. HIV infection is a chronic infection that persists in lymphoid tissues even in the context of antiretroviral therapy causing profound tissue-specific alterations (15, 55, 78–80). This characteristic of HIV pathogenesis has prompted several cure strategies aiming at viral reservoir elimination to be developed over the past years (81). In this context, we designed a panel that leverages the analytical strength of the RNAscope *in situ* hybridization platform (ACD) and resolving power of confocal microscopy for topological examination and quantification of HIV-specific RNA in tissues. RNAscope is a widely used methodology that employs RNA probe hybridization and a system of cascading fluorescent probes to detect viral RNA, a surrogate of actively transcribing virus, *in situ* with high specificity and signal-to-noise resolution (82, 83). In this application, viral RNA detection is extended to include relevant immune cell populations of interest by staining fluorescently for CD3, CD4 and FDC (**Figure 8A**). A nuclear stain is also used to allow for individual cell segmentation whilst the specificity of the vRNA+ signal in the assay was ascertained through RNAse -mediated signal elimination in a control experiment (**Supplementary Figure 3B**). We use this panel to map virus RNA expression based on the RNAscope signal and quantify infected cells in intra-follicular (CD4^lo^CD3^lo^) as well as extra-follicular (CD4^hi^CD3^hi^) locations using HistoCytometry as well as FDC-bound virions using Spots analysis (**Figure 8B**). Analysis of a lymph node tissue section from an HIV+ individual revealed a trend for higher numbers of FDC-bound virions in follicles with lower overall frequencies of CD4^hi/dim^ T-cells (**Figure 8C**). Follicular (F) spaces harbored a higher proportion of vRNA^+^ CD4 T cells compared to EF spaces (**Figure 8D**, pie charts) however the frequency of CD4^dim^ T cells harboring actively transcribing virus was higher in EF compared with F areas in the absence of therapy. In addition, within F areas, higher levels of vRNA+ signal were observed in CD3^hi^CD4^dim^ T cells compared to CD3^hi^CD4^hi^ T cells (**Figure 8D**). This approach thus offers an integrative way to study tissue-specific viral burden and kinetics in relation to T cell immune dynamics at different infection stages or after the initiation of therapy.

## Discussion

The integration of lymphoid tissue analysis in studies of infection and vaccination can offer important insights into the mechanisms underlying disease progression or protection. In this study, we describe the development of a pipeline of eight multispectral confocal panels that can be used to track a multitude of immune cell subsets *in situ*, both innate and adaptive, in relevant lymphoid tissue localities for mechanistic insights and hypothesis-driven experimentation (**Supplementary Figure 1B**). The adoption of laser-scanning confocal microscopy (LSCM) for image acquisition offers many advantages over traditional widefield methodologies including high lateral and axial spatial resolution as well as capacity for volumetric 3D analysis and high multiplexing (84), especially when iterative staining protocols, such as CyCIF are employed (85). Even though in this study we used tissues with a thickness of ~10um, thicker tissues have successfully been imaged by us and others using immunofluorescence and confocal microscopy (24, 86). To allow for increased accuracy in downstream image segmentation we imaged tissues at 40x magnification and recorded between 40000 and 70000 individual cells. Furthermore, accurate representation and minimization of selection bias was ensured by imaging at least 50% of the tissue or by capturing 5 follicles on average in line with previously published research in our lab (24, 30) We also opted to repeat selected markers among the different panels to address sampling errors arising from the analysis of a single tissue, measured populations with and without area normalization and examined key tonsillar B cell and Tfh cell populations by flow cytometry to further confirm the validity of our tissue analysis observations (**Supplementary Figure 1A**). Wherever possible, we used previously validated antibodies in our assays. For antibodies not previously tested with FFPE tissues specificity was confirmed by i) checking the staining localization with respect to the nuclear staining, ii) studying the localization of the staining on the tissue iii) confirming the co-localization profile on lineage markers and iv) by including wherever possible an isotype control (**Supplementary Figure 3C**). Image acquisition was performed in a microscope fitted with 11 lasers. Spill-over among the various channels was minimized by: i) carefully selecting the spectral areas corresponding to each fluorophore after testing each fluorophore separately to verify the exact emission curve (**Supplementary Figures 4A, B**), ii) titrating the antibodies for optimal signal, iii) collecting fluorophore emission on separate detectors with fluorophores being excited sequentially (2-3 fluorophores/sequential, and iv) creating a spillover compensation matrix using singly stained samples and applying it to the collected dataset. In our applications autofluorescence-specific background elimination was not necessary for the most part as the validated antibodies gave excellent signal-to-noise ratios after titration. However, when this was of relevance autofluorescence was eliminated by recording the autofluorescent signal in a separate channel and subtracting it from all other channels post-acquisition or for mutually exclusive markers only, through paired subtraction. On the Imaris software this can be achieved through the channel arithmetics module. We used 8 lasers to acquire our images (**Supplementary Figure 4A**) but instruments with fewer lasers can also be used. In this case, spectrally neighboring fluorescent stains (i.e. Alexa Fluor 647 and Alexa Fluor 700) can be acquired with live spectral unmixing under a single laser. In such a scenario antibody titration is highly recommended for optimal live spectral unmixing as the intensity of the staining may vary depending on target protein expression levels and the staining protocol used. Even though our study did not make use of iterative staining protocols such as Cy-CIF (85), evaluation of such protocols in future studies is warranted as a means to increase dimensionality and maximize the use of tissue specimens with low availability. Our panels employ a total of 28 individual protein markers used to characterize ~8 distinct immune and stromal cell lineages (CD20, CD4, CD8, Neutrophils, Monocytes, FDCs, BECs/LECs, DCs) or ~ 32 distinct immune subpopulations. Staining in tandem could thus increase the dimensionality and throughput of proposed single-cell analyses to >30 distinct marker combinations. To demonstrate application in seven out of eight panels (GC reactivity; T-cell regulation; B-cell immunity; CD8 T-cell positioning and function; monocyte and inflammatory markers; GC microarchitecture; and tissue-specific DC subpopulations), we chose to focus on tonsillar tissues and defined B-cell follicles either as CD20^hi/dim^ or as GCs (CD20^hi/dim^ Ki67^+^) (**Supplementary Figure 1C**). We found that each definition has its own merits in terms of quantification but selection of either for analysis should be carefully considered as distinct topologies may be associated with distinct functions and phenotypes. We chose tonsillar tissue because this type of tissue is easier to obtain compared to resting, HIV negative LNs. In addition, tonsillar tissue represents a chronically inflamed tissue exhibiting a highly organized stereotypical microanatomy compared to LNs and as such it is ideal for the study of tissue resident populations connected to activation and secondary B-cell follicle formation, such as monocytes and Tfh (30). In addition, previous research in our lab has shown that HIV negative tonsils and HIV negative LNs share several common topological microanatomical attributes including i) common secondary B-cell follicle microarchitecture (MZ, LZ, DZ); ii) common GC (CD20^hi^Ki67^hi^) polarization, and iii) comparable aspects of Tfh heterogeneity and polarization (24). LNs on the other hand can acquire divergent phenotypes in states of pathology, as in the case of HIV infection, that complicate the interpretation of physiological microanatomical boundaries (87). Even though discussion of LN-associated pathology extends beyond the scope of this article, the application of the panels to different tissue preparations is warranted. This is especially informative for infections that persist chronically by establishing life-long reservoirs in the tissues of infected hosts. The detection and persistence of a pathogen in tissues can be address using a number of different methodologies. These range, depending on the size of the pathogen, from direct microscopic examination to detection of pathogen-associated proteins using monoclonal antibodies (88) or associated DNA/RNA using molecular probes (89). In the case of HIV infection in particular, one of the main challenges that need to be overcome for cure is the presence of tissue-specific reservoirs that persist in infected individuals for life seeding viral replication (78, 90). Tissue analysis and *in situ* tracking of viral RNA can help address several key questions that remain in this field. For example, what is the *in vivo* phenotype of cells harboring actively transcribing virus or latent infection? Where do these cells localize and what are the micro-geographical patterns or cell-to-cell interactions that enable persistence? Does tissue-specificity modulate the function and phenotype of latently infected cells? Are latency and persistence associated with distinct non-immune tissue-specific biomarkers (i.e. stromal cells) or soluble factor distributions *in situ* (i.e pro-inflammatory chemokines and cytokines)? Tissue analysis also offers a tangible way to measure the effect of latency reversal interventions and a means to assess the reactogenicity and immunogenicity of novel vaccine formulations. Confocal imaging in particular, with its high resolution and volumetric capacity, offers an ideal platform for the comprehensive mapping of protein markers at subcellular level.

In summary, we have described a detailed tissue analysis pipeline that can be used to characterize geographical patterns, immune-cell distributions and relationships in lymphoid tissues. Application of this type of analysis can increase the dimensionality and predictive power of flow-cytometry and single-cell RNA expression analyses and accelerate biomarker discovery in the context of infection and vaccination.

## Data Availability Statement

The original contributions presented in the study are included in the article/**Supplementary Material**. Further inquiries can be directed to the corresponding author.

## Ethics Statement

The studies involving human participants were reviewed and approved by INER-CIENI Ethics Committee. The patients/participants provided their written informed consent to participate in this study.

## Author Contributions 

EM, CP, and RK conceived and designed the study. PR, FT-R, and GR-T collected samples. EM performed the experiments, undertook the analysis and wrote the manuscript. CP and RK supervised the study. All authors contributed to the article and approved the submitted version.

## Funding

This research was supported by the Intramural Research Program of the Vaccine Research Center, NIAID, National Institutes of Health; and a CAVD grant (#OP1032325) from the Bill and Melinda Gates Foundation.

## Conflict of Interest

The authors declare that the research was conducted in the absence of any commercial or financial relationships that could be construed as a potential conflict of interest.

## Supplementary Material

Supplementary Figure 1 Quantitation of relevant immune cell populations using HistoCytometry and Flow Cytometry, confocal imaging method overview and positional heterogeneity of CD4+ T cell subsets in tonsillar B-cell follicles. **(A)** Box plots showing the relative frequencies of CD4 T cells, CD20 and CD20^+^Ki67^+^ B cells as well as CD8 T cells in tonsillar sections as well as the cell numbers for each population normalized by the total corresponding tissue area scanned (mm3). B cells (CD20+) were the most abundant population in tonsils (median frequency 35%) followed by CD4 T cells (median frequency 27%) whilst the frequency of CD8 T cells was relatively low (7-9.5% of total cells). Higher ratios (CD20+ cells/mm^3^) represent tissues with higher B-cell (CD20+) abundance. The frequencies of the corresponding tonsillar populations as determined by flow cytometry (n=6) are also shown. **(B)** Schematic illustrating the methodology used for the acquisition and analysis of the eight multispectral confocal datasets. (1) Tissues are stained with pre-titrated combinations of antibodies (2) and images are acquired on a laser scanning confocal microscope (Leica TCS SP8) using a 40x (NA 1.3) objective at 512x512 pixel density and 1% zoom. (3) Image segmentation for single cells is performed with the program Imaris based on the nuclear stain (JOPRO-1) and associated surface marker intensities and areas are then exported for graphing (4a) or combined with X-Y positional information (4b) and exported to excel to create a csv file for FlowJo import (5) and further gating and analysis (6). **(C)** (Left)Representative confocal microscopy image of a tonsillar B cell follicle as determined by immunofluorescent staining against the B cell marker CD20 (cyan) and the DZ/LZ specific marker Ki67 (magenta). White enclosures denote the boundaries of the T zone (T), follicle (F), marginal zone (MZ) and germinal center (GC) (Middle) Bar graph summarizing the frequencies of PD-1^hi^ CD4+ T-cells in the area surrounding a B-cell follicle (T-zone), as well as within the F, MZ and GC. Each circle represents an individual follicle (n=6) and data were obtained from the analysis of a single tonsil tissue section (n=1) (Right) Confocal microscopy images showing the CD4 T cell populations corresponding to each individual B-cell follicle locality as determined by the distribution of the CD4 (green) and PD-1 (red). For visualization purposes MZ and GC images were masked post-acquisition on Imaris to only show the cells that belong to the area indicated. Tfh are identified by the co-expression of CD4 (green) and PD-1 (red). (D) Gating strategy used for the characterization of B-cell and Tfh populations (CXCR5^hi^PD-1^hi^CD57^+/-^) using flow cytometry. Images were acquired at 40x (NA 1.3) with 1% zoom. Scale bars = 100um and 10um (close-ups).

Supplementary Figure 2 Patterns of H2Ax staining, enumeration of CD3-CD8+ NK cells in tonsils and alternative CD8+ T cell imaging panel. **(A)** Representative confocal images showing the distribution of pH2Ax in GCs after staining for pH2Ax (red), CD20 (cyan) and Ki67 (magenta) (left) and representative gating strategy used for the characterization of the frequencies of pH2Ax^hi^ expression in CD20^dim^CD38^lo^, CD20^hi^CD38^dim^ and CD20^lo^CD38^hi^ B cells in a flow cytometry experiment. The frequencies of pH2Ax/Volume^hi^ events in F and GCs as measured by confocal imaging and HistoCytometry in two tonsils (n=2). and two HIV+ LN (n=2) are summarized in the bar graph shown. Circles represent individual follicles whilst different specimens are demarcated by individual colors. **(B)** Gating strategy used for the flow cytometrical characterization of CD8+ CD3- T cells in tonsils and histogram depicting the proportion of CD3^+^ and CD3^-^ cells within the CD8^bright^ and CD8^dim^ gates. A summary of the relative frequencies of CD8^bright^CD3^-^ cells in five tonsils is given in the bar graph shown. **(C)** Alternative CD8+ T cell confocal imaging panel combining the markers CD3 (purple), CD8 (red), FasL (yellow) and GrzB (orange) for the characterization of CD3^+^CD8^+^ and CD3^-^CD8^+^ lymphocytes in lymphoid tissues where higher NK frequencies are expected and b) relative frequencies of CD3+CD8+ and CD3-CD8+ populations as measured by histocytometry in one tonsillar tissue and one HIV+ LN after application of the alternative CD8 T cell imaging panel. Images were acquired at 40x (NA 1.3) with 1% zoom. Scale bars are 100um, 50um and 10um.

Supplementary Figure 3 CD8+ FasL^+/-^ GrzB ^+/-^ T cells in tonsils and LNs, IF-FISH RNAscope staining controls and fluorescence minus one (FMO) controls. **(A)** Histocytometry plots showing the distribution of imaged follicles in one tonsillar and one HIV+ LN tissue as denoted by CD20 (cyan) and Ki67 (magenta) staining and the gating strategy used for the identification of CD8+ FasL GrzB expressing populations within these localities. Red circles denote individual follicles. The cumulative frequencies of CD8+ Fas^L+/-^ GrzB ^+/-^ T cells for all follicles measured in the two types of samples are shown in the bar graph. Each type of sample is denoted by a different colour. **(B)** Confocal microscopy images showing two sequential LN tissue sections from an HIV+ individual stained with an HIV+ vRNA+ specific probe and antibodies specific for the protein markers CD3 (cyan) CD4 (magenta) and FDC (green) in the absence (section 1) or presence (section 2) of an RNase A/T1 pretreatment step. The localization of the vRNA+ signal (red) on the FDC network (green) is shown. **(C)** Representative example of FMO controls used to establish the specificity of the detected signals in each channel. Fluorescence in the FMO channel was detected only when the corresponding antibody was present but not when omitted or replaced by an isotype control. Images were acquired at 40x (NA 1.3) with 1% zoom. Scale bars = 500um, 50um (close-ups).

Supplementary Figure 4 Fluorescence acquisition spectral regions and cross-talk among acquisition channels. **(A)** Schematic showing the lasers, fluorochromes used and photon detection regions used for image acquisition in the eight multispectral panels described in this study. Regions were adjusted after acquiring real-time single fluorochrome emission curves to maximize signal-to-noise ratios and minimize cross-talk between channels. **(B)** Representative example of single fluorochrome emission cross-over into neighboring channels as detected after staining for BV421, JOPRO-1, eF615, AF488, AF546, AF647, BV480 or AF700. Each horizontal line corresponds to a single fluorochrome that was acquired under the same conditions as the full panel in order to visualize and resolve potential fluorescence cross-over issues. Images were acquired at 40x (NA 1.3) with 1% zoom. Scale bars are 100um.
